# Poly[[tetra­aqua­bis­(μ_3_-5-carboxybenzene-1,2,4-tri­carboxyl­ato)tricadmium] tetra­hydrate]

**DOI:** 10.1107/S1600536812022726

**Published:** 2012-05-26

**Authors:** Yong-Yan Jia, Xin-Nian Xie, Huai-Xia Yang

**Affiliations:** a Pharmacy College, Henan University of Traditional Chinese Medicine, Zhengzhou 450008, People’s Republic of China

## Abstract

There are three independent Cd^II^ ions in the title complex, {[Cd_3_(C_10_H_3_O_8_)_2_(H_2_O)_4_]·4H_2_O}_*n*_, one of which is coordinated by four O atoms from three 5-carboxybenzene-1,2,4-tri­carboxyl­ate ligands and by two water mol­ecules in a distorted octa­hedral geometry. The second Cd^II^ ion is coordinated by five O atoms from four 5-carboxybenzene-1,2,4-tri­carboxyl­ate ligands and by one water mol­ecule also in a distorted octa­hedral geometry while the third Cd^II^ ion is coordinated by five O atoms from three 5-carboxybenzene-1,2,4-tri­carboxyl­ate ligands and by one water mol­ecule in a highly distorted octa­hedral geometry. The 5-carboxybenzene-1,2,4-tri­carboxyl­ate ligands bridge the Cd^II^ ions, resulting in the formation of a three-dimensional structure. Intra- and inter­molecular O—H⋯O hydrogen bonds are present throughout the three-dimensional structure.

## Related literature

For background information on Cd^II^ complexes constructed from benzene-1,2,4,5-tetra­carb­oxy­lic acid ligand see: Lin *et al.* (2008 ▶); Prajapati *et al.* (2009 ▶); Wang *et al.* (2012 ▶). 
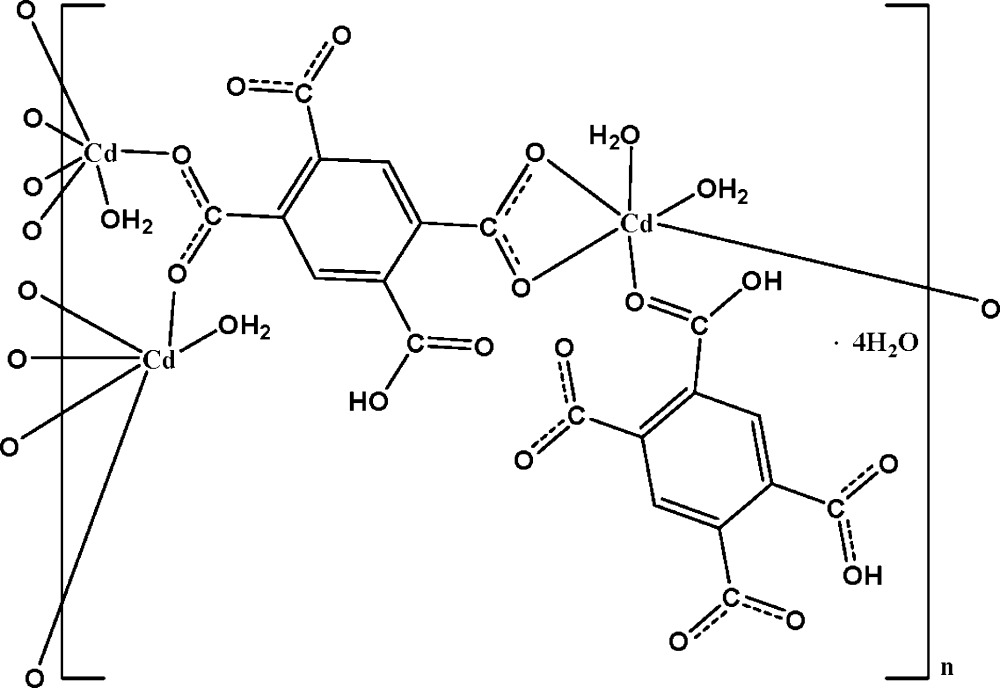


## Experimental

### 

#### Crystal data

[Cd_3_(C_10_H_3_O_8_)_2_(H_2_O)_4_]·4H_2_O
*M* *_r_* = 983.58
Triclinic, 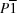

*a* = 8.3244 (17) Å
*b* = 12.992 (3) Å
*c* = 13.540 (3) Å
α = 85.79 (3)°
β = 84.67 (3)°
γ = 87.17 (3)°
*V* = 1452.6 (5) Å^3^
*Z* = 2
Mo *K*α radiation
μ = 2.28 mm^−1^
*T* = 293 K
0.20 × 0.17 × 0.16 mm

#### Data collection

Rigaku Saturn diffractometer
Absorption correction: multi-scan (*CrystalClear*; Rigaku/MSC, 2004 ▶) *T*_min_ = 0.658, *T*_max_ = 0.712
18101 measured reflections
6873 independent reflections
6308 reflections with *I* > 2σ(*I*)
*R*_int_ = 0.022

#### Refinement

*R*[*F* ^2^ > 2σ(*F* ^2^)] = 0.029
*wR*(*F* ^2^) = 0.068
*S* = 1.05
6873 reflections
424 parameters
H-atom parameters constrained
Δρ_max_ = 0.64 e Å^−3^
Δρ_min_ = −0.84 e Å^−3^

### 

Data collection: *CrystalClear* (Rigaku/MSC, 2004 ▶); cell refinement: *CrystalClear*; data reduction: *CrystalClear*; program(s) used to solve structure: *SHELXS97* (Sheldrick, 2008 ▶); program(s) used to refine structure: *SHELXL97* (Sheldrick, 2008 ▶); molecular graphics: *SHELXTL* (Sheldrick, 2008 ▶); software used to prepare material for publication: *publCIF* (Westrip, 2010 ▶).

## Supplementary Material

Crystal structure: contains datablock(s) global, I. DOI: 10.1107/S1600536812022726/lh5477sup1.cif

Structure factors: contains datablock(s) I. DOI: 10.1107/S1600536812022726/lh5477Isup2.hkl

Additional supplementary materials:  crystallographic information; 3D view; checkCIF report

## Figures and Tables

**Table 1:** Hydrogen-bond geometry (Å, °)

*D*—H⋯*A*	*D*—H	H⋯*A*	*D*⋯*A*	*D*—H⋯*A*
O11—H11⋯O22	0.82	2.16	2.895 (4)	149
O17—H17*C*⋯O8	0.84	2.32	3.158 (4)	179
O18—H18*B*⋯O3	0.85	2.50	3.292 (4)	156
O2—H2⋯O10^i^	0.82	2.53	3.237 (3)	145
O18—H18*A*⋯O10^ii^	0.85	2.19	3.031 (4)	168
O24—H24*B*⋯O15^iii^	0.85	2.09	2.841 (4)	147
O19—H19*A*⋯O23^iv^	0.85	1.92	2.720 (4)	157
O17—H17*B*⋯O23^v^	0.85	2.29	3.073 (5)	155
O19—H19*B*⋯O6^vi^	0.85	1.85	2.698 (3)	173
O20—H20*B*⋯O6^vi^	0.85	2.18	2.996 (4)	160
O20—H20*C*⋯O13^vii^	0.85	2.23	3.047 (4)	161
O22—H22*A*⋯O2^vii^	0.85	2.05	2.807 (4)	147
O23—H23*B*⋯O7^vii^	0.85	2.09	2.894 (4)	158
O24—H24*A*⋯O4^viii^	0.85	1.94	2.783 (4)	173
O21—H21*A*⋯O9^ix^	0.85	1.91	2.748 (4)	168
O21—H21*B*⋯O12^x^	0.85	1.94	2.765 (4)	163
O22—H22*B*⋯O21^xi^	0.85	1.99	2.825 (4)	166
O23—H23*A*⋯O2^xii^	0.85	2.20	2.944 (4)	147

Symmetry codes: (i) 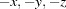; (ii) 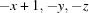; (iii) 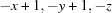; (iv) 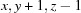; (v) 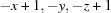; (vi) 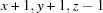; (vii) 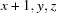; (viii) 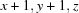; (ix) 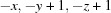; (x) 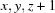; (xi) 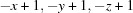; (xii) 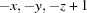.

**Fig. 1.**
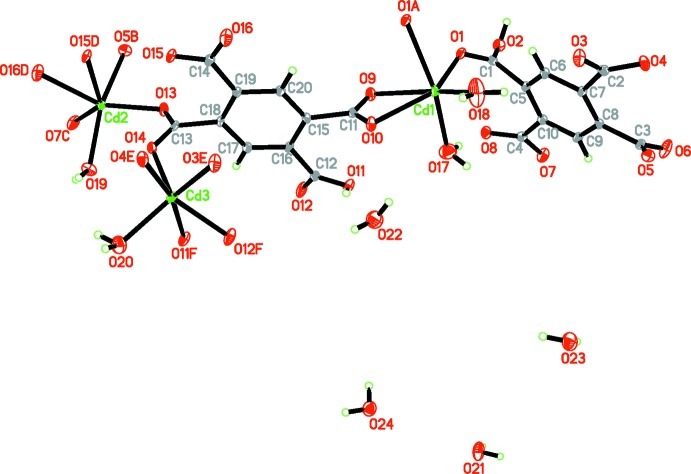
View of the title complex showing labeling and 30% probability displacement ellipsolids. [Symmetry code A: -x, -y, -z, B: x, y + 1, z - 1; C: -x, -y + 1, -z; D: -x + 1, -y + 1, -z - 1; E: -x + 1, -y, -z; F: -x + 1, -y + 1, -z.].

**Fig. 2.**
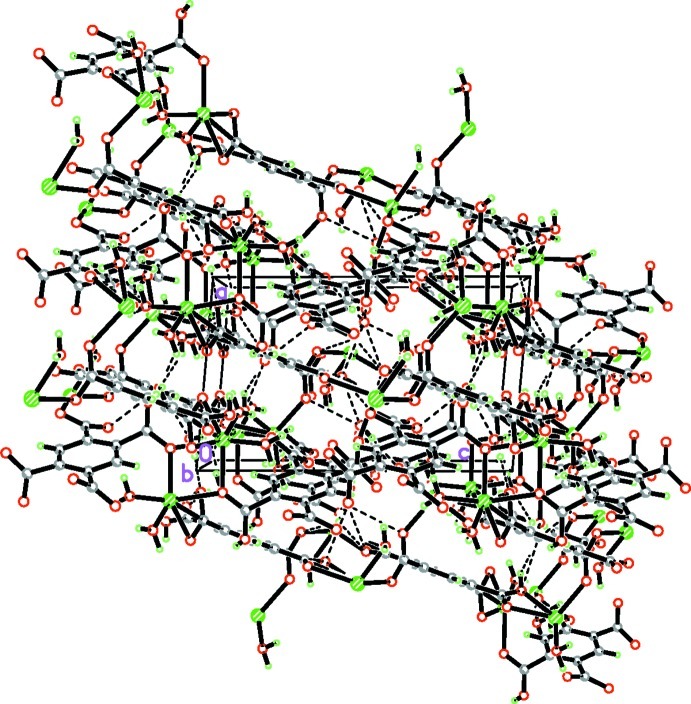
Packing plot of the title complex with hydrogen bonds indicated by dashed lines.

## supplementary crystallographic information

### Comment

A large number of Cd^II^ complexes constructed from benzene-1,2,4,5-tetracarboxylic acid have been extensively studied because of the diversity coordination modes and sensitivity to pH values of the carboxylate groups. Some of the final products exhibit useful functional properties (Lin *et al.*, 2008; Prajapati *et al.*, 2009; Wang *et al.*, 2012). In order to further explore such compounds with new structures, we selected benzene-1,2,4,5-tetracarboxylic acid as ligand to self-assembly with Cd(NO_3_)_2_ and obtained the title complex, {[Cd_3_(C_10_H_3_O_8_)_2_(H_2_O)_4_] (H_2_O)_4_}_n_, the crystal structure of which is reported herein. As shown in Fig. 1, there are three crystallographically independent cadmium ions (Cd1, Cd2 and Cd3), two crystallographically independent 5-carboxybenzene-1,2,4-tricarboxylate ligands, four crystallographically independent coordination water molecules and four crystallographically independent solvent water molecules in the asymmetric unit. Atom Cd1 displays a distorted octahedral geometry defined by atoms O1, O9, O10 from two 5-carboxybenzene-1,2,4-tricarboxylate groups and O18 from water molecule in equatorial positions and by atoms O1A, O17 from one 5-carboxybenzene-1,2,4-tricarboxylate group and one water molecule in axial positions (symmetry codes A to F are given in the figure caption). Atom Cd2 is coordinated by six oxygen atoms from four 5-carboxybenzene-1,2,4-tricarboxylate groups (O5B, O7C, O13, O15D, O16D) and one water molecule (O19) leading to a distorted octahedral geometry. Atom Cd3 is coordinated by five O atoms (O3E, O4E, O11F, O12F, O14) from three 5-carboxybenzene-1,2,4-tricarboxylate groups and by one O atom (O20) from water molecule in a seriously distorted octahedral geometry. As depicted in Fig. 2, Cd1, Cd2 and Cd3 ions are bridged by 5-carboxybenzene-1,2,4-tricarboxylate ligands forming the three-dimensional structure in which the carboxylate groups of the 5-carboxybenzene-1,2,4-tricarboxylate ligands coordinate to Cd^II^ ions in monodentate mode, or in chelating mode or in bridging mode. In addition, intramolecular O—H···O hydrogen bonds between the coordinated water molecules and carboxylate groups stabilize the molecular conformation. In the crystal, O—H···O hydrogen bonds (see Table 1 for full listing) form a three-dimensional network throughout the polymer structure (Fig. 2).

### Experimental

A mixture of Cd(NO_3_)_2_ (0.05 mmol), benzene-1,2,4,5-tetracarboxylic acid (0.05 mmol),water (4 ml) and methanol(4 ml) was placed in a 25 ml Teflon-lined stainless steel vessel and heated at 293K for 72 h, then cooled to room temperature. Colourless crystals were obtained from the filtrate and dried in air.

### Refinement

H atoms bound to C atoms were positioned geometrically and refined as riding atoms, with C-H = 0.93 Å. H atoms bound to O atoms were found from difference maps and included with O—H = 0.82—0.85Å All H atoms were refined with U_iso_(H) = 1.2 U_eq_(C,O).

### Crystal data

**Table:**

[Cd_3_(C_10_H_3_O_8_)_2_(H_2_O)_4_]·4H_2_O	*Z* = 2
*M**_r_* = 983.58	*F*(000) = 956
Triclinic, *P*1	*D*_x_ = 2.249 Mg m^−^^3^
*a* = 8.3244 (17) Å	Mo *K*α radiation, λ = 0.71073 Å
*b* = 12.992 (3) Å	Cell parameters from 4891 reflections
*c* = 13.540 (3) Å	θ = 1.6–27.9°
α = 85.79 (3)°	µ = 2.28 mm^−^^1^
β = 84.67 (3)°	*T* = 293 K
γ = 87.17 (3)°	Prism, colourless
*V* = 1452.6 (5) Å^3^	0.20 × 0.17 × 0.16 mm

### Data collection

**Table:**

Rigaku Saturn diffractometer	6873 independent reflections
Radiation source: fine-focus sealed tube	6308 reflections with *I* > 2σ(*I*)
Graphite monochromator	*R*_int_ = 0.022
Detector resolution: 28.5714 pixels mm^-1^	θ_max_ = 27.9°, θ_min_ = 1.5°
ω scans	*h* = −10→10
Absorption correction: multi-scan (*CrystalClear*; Rigaku/MSC, 2004)	*k* = −17→16
*T*_min_ = 0.658, *T*_max_ = 0.712	*l* = −17→17
18101 measured reflections	

### Refinement

**Table:**

Refinement on *F*^2^	Primary atom site location: structure-invariant direct methods
Least-squares matrix: full	Secondary atom site location: difference Fourier map
*R*[*F*^2^ > 2σ(*F*^2^)] = 0.029	Hydrogen site location: inferred from neighbouring sites
*wR*(*F*^2^) = 0.068	H-atom parameters constrained
*S* = 1.05	*w* = 1/[σ^2^(*F*_o_^2^) + (0.031*P*)^2^ + 2.6215*P*] where *P* = (*F*_o_^2^ + 2*F*_c_^2^)/3
6873 reflections	(Δ/σ)_max_ = 0.002
424 parameters	Δρ_max_ = 0.64 e Å^−^^3^
0 restraints	Δρ_min_ = −0.84 e Å^−^^3^

### Special details

**Table:**

Geometry. All e.s.d.'s (except the e.s.d. in the dihedral angle between two l.s. planes) are estimated using the full covariance matrix. The cell e.s.d.'s are taken into account individually in the estimation of e.s.d.'s in distances, angles and torsion angles; correlations between e.s.d.'s in cell parameters are only used when they are defined by crystal symmetry. An approximate (isotropic) treatment of cell e.s.d.'s is used for estimating e.s.d.'s involving l.s. planes.
Refinement. Refinement of *F*^2^ against ALL reflections. The weighted *R*-factor *wR* and goodness of fit *S* are based on *F*^2^, conventional *R*-factors *R* are based on *F*, with *F* set to zero for negative *F*^2^. The threshold expression of *F*^2^ > σ(*F*^2^) is used only for calculating *R*-factors(gt) *etc*. and is not relevant to the choice of reflections for refinement. *R*-factors based on *F*^2^ are statistically about twice as large as those based on *F*, and *R*- factors based on ALL data will be even larger.

### Fractional atomic coordinates and isotropic or equivalent isotropic displacement parameters (Å^2^)

**Table:**

	*x*	*y*	*z*	*U*_iso_*/*U*_eq_	
Cd1	0.16008 (3)	0.030815 (17)	0.077320 (17)	0.02133 (6)	
Cd2	0.38339 (3)	0.741101 (16)	−0.452927 (15)	0.01619 (6)	
Cd3	0.82987 (3)	0.508576 (17)	−0.221027 (15)	0.01826 (6)	
O1	−0.1133 (3)	0.01484 (17)	0.09031 (15)	0.0218 (5)	
O2	−0.3553 (3)	0.00697 (18)	0.17445 (17)	0.0253 (5)	
H2	−0.3953	−0.0381	0.1465	0.030*	
O3	0.1589 (3)	−0.33132 (18)	0.21984 (18)	0.0309 (6)	
O4	0.0193 (3)	−0.40654 (16)	0.34811 (17)	0.0257 (5)	
O5	0.1396 (3)	−0.30563 (17)	0.50753 (18)	0.0240 (5)	
O6	−0.1027 (3)	−0.2697 (2)	0.58004 (18)	0.0371 (6)	
O7	−0.2938 (3)	0.08991 (18)	0.46215 (17)	0.0303 (6)	
O8	−0.2171 (3)	0.14979 (17)	0.30916 (17)	0.0256 (5)	
O9	0.1276 (3)	0.19607 (18)	−0.00883 (17)	0.0239 (5)	
O10	0.3778 (3)	0.13809 (17)	0.00776 (17)	0.0239 (5)	
O11	0.2987 (3)	0.35204 (18)	0.10793 (16)	0.0259 (5)	
H11	0.3902	0.3256	0.1010	0.031*	
O12	0.2293 (4)	0.50979 (18)	0.05480 (17)	0.0332 (6)	
O13	0.3761 (3)	0.59828 (17)	−0.33999 (16)	0.0216 (5)	
O14	0.6352 (3)	0.56376 (16)	−0.31637 (16)	0.0196 (4)	
O15	0.5157 (3)	0.39054 (17)	−0.43313 (15)	0.0219 (5)	
O16	0.5220 (4)	0.22733 (19)	−0.37873 (19)	0.0388 (7)	
O17	0.1438 (4)	0.1222 (3)	0.2170 (2)	0.0504 (8)	
H17B	0.1858	0.0889	0.2650	0.060*	
H17C	0.0472	0.1294	0.2412	0.060*	
O18	0.3002 (4)	−0.1137 (3)	0.1143 (3)	0.0722 (12)	
H18A	0.3966	−0.1163	0.0873	0.087*	
H18B	0.2859	−0.1668	0.1542	0.087*	
O19	0.5894 (3)	0.79789 (18)	−0.37472 (19)	0.0298 (5)	
H19A	0.5794	0.8634	−0.3773	0.036*	
H19B	0.6889	0.7805	−0.3857	0.036*	
O20	1.0312 (3)	0.6158 (2)	−0.2434 (2)	0.0431 (7)	
H20B	1.0047	0.6608	−0.2884	0.052*	
H20C	1.1237	0.5955	−0.2685	0.052*	
O21	0.1511 (4)	0.6937 (2)	0.9506 (2)	0.0476 (8)	
H21A	0.0610	0.7199	0.9737	0.057*	
H21B	0.1606	0.6418	0.9920	0.057*	
O22	0.5749 (4)	0.2208 (2)	0.1579 (2)	0.0488 (8)	
H22A	0.5693	0.1581	0.1450	0.059*	
H22B	0.6664	0.2381	0.1301	0.059*	
O23	0.6103 (4)	0.0002 (2)	0.6595 (2)	0.0484 (8)	
H23A	0.5277	0.0223	0.6948	0.058*	
H23B	0.6288	0.0415	0.6084	0.058*	
O24	0.7826 (3)	0.5204 (2)	0.4913 (2)	0.0378 (6)	
H24A	0.8507	0.5474	0.4476	0.045*	
H24B	0.6930	0.5225	0.4656	0.045*	
C1	−0.2069 (4)	−0.0074 (2)	0.1688 (2)	0.0171 (6)	
C2	0.0634 (4)	−0.3266 (2)	0.2965 (2)	0.0179 (6)	
C3	0.0021 (4)	−0.2601 (2)	0.5089 (2)	0.0176 (6)	
C4	−0.2286 (4)	0.0777 (2)	0.3753 (2)	0.0186 (6)	
C5	−0.1338 (3)	−0.0616 (2)	0.2577 (2)	0.0152 (5)	
C6	−0.0544 (3)	−0.1566 (2)	0.2429 (2)	0.0163 (6)	
H6A	−0.0320	−0.1770	0.1784	0.020*	
C7	−0.0074 (3)	−0.2223 (2)	0.3227 (2)	0.0153 (5)	
C8	−0.0389 (3)	−0.1918 (2)	0.4193 (2)	0.0150 (5)	
C9	−0.1116 (4)	−0.0943 (2)	0.4335 (2)	0.0171 (6)	
H9A	−0.1293	−0.0727	0.4977	0.021*	
C10	−0.1586 (3)	−0.0284 (2)	0.3547 (2)	0.0162 (6)	
C11	0.2780 (4)	0.2045 (2)	−0.0247 (2)	0.0174 (6)	
C12	0.2843 (4)	0.4197 (2)	0.0387 (2)	0.0179 (6)	
C13	0.4879 (3)	0.5440 (2)	−0.3039 (2)	0.0142 (5)	
C14	0.4981 (4)	0.3204 (2)	−0.3644 (2)	0.0184 (6)	
C15	0.3382 (3)	0.2933 (2)	−0.0935 (2)	0.0154 (5)	
C16	0.3343 (4)	0.3948 (2)	−0.0658 (2)	0.0168 (6)	
C17	0.3824 (4)	0.4731 (2)	−0.1368 (2)	0.0179 (6)	
H17A	0.3779	0.5409	−0.1186	0.021*	
C18	0.4369 (3)	0.4528 (2)	−0.2338 (2)	0.0144 (5)	
C19	0.4427 (3)	0.3504 (2)	−0.2610 (2)	0.0155 (5)	
C20	0.3926 (4)	0.2723 (2)	−0.1903 (2)	0.0164 (6)	
H20A	0.3956	0.2045	−0.2085	0.020*	

### Atomic displacement parameters (Å^2^)

**Table:**

	*U*^11^	*U*^22^	*U*^33^	*U*^12^	*U*^13^	*U*^23^
Cd1	0.02465 (12)	0.01847 (12)	0.02003 (11)	−0.00301 (9)	−0.00097 (9)	0.00440 (8)
Cd2	0.02026 (11)	0.01366 (11)	0.01385 (10)	0.00249 (8)	0.00120 (8)	−0.00115 (8)
Cd3	0.02283 (11)	0.01690 (11)	0.01451 (10)	0.00382 (8)	0.00031 (8)	−0.00328 (8)
O1	0.0248 (11)	0.0261 (12)	0.0142 (10)	−0.0037 (9)	−0.0006 (9)	0.0023 (9)
O2	0.0191 (11)	0.0274 (12)	0.0292 (12)	0.0018 (9)	−0.0038 (9)	0.0004 (10)
O3	0.0424 (14)	0.0185 (12)	0.0276 (12)	0.0088 (10)	0.0142 (11)	−0.0024 (10)
O4	0.0343 (13)	0.0126 (10)	0.0281 (12)	0.0004 (9)	0.0075 (10)	−0.0012 (9)
O5	0.0196 (11)	0.0203 (11)	0.0327 (12)	0.0063 (9)	−0.0082 (9)	−0.0023 (9)
O6	0.0302 (13)	0.0521 (17)	0.0235 (12)	0.0133 (12)	0.0058 (10)	0.0142 (12)
O7	0.0465 (15)	0.0221 (12)	0.0201 (11)	0.0132 (11)	0.0041 (10)	−0.0052 (9)
O8	0.0343 (13)	0.0148 (11)	0.0267 (12)	0.0031 (9)	−0.0031 (10)	0.0025 (9)
O9	0.0209 (11)	0.0248 (12)	0.0245 (11)	−0.0038 (9)	0.0012 (9)	0.0055 (9)
O10	0.0254 (11)	0.0192 (11)	0.0248 (12)	0.0027 (9)	0.0030 (9)	0.0058 (9)
O11	0.0337 (13)	0.0269 (12)	0.0150 (10)	0.0087 (10)	0.0025 (9)	0.0009 (9)
O12	0.0628 (18)	0.0184 (12)	0.0154 (11)	0.0084 (11)	0.0076 (11)	−0.0019 (9)
O13	0.0201 (11)	0.0199 (11)	0.0227 (11)	0.0018 (8)	0.0002 (9)	0.0082 (9)
O14	0.0181 (10)	0.0206 (11)	0.0192 (10)	−0.0029 (8)	0.0002 (8)	0.0031 (8)
O15	0.0264 (11)	0.0247 (12)	0.0125 (10)	0.0026 (9)	0.0036 (8)	0.0025 (8)
O16	0.0687 (19)	0.0186 (12)	0.0254 (13)	0.0009 (12)	0.0180 (12)	−0.0073 (10)
O17	0.0495 (18)	0.062 (2)	0.0403 (17)	−0.0057 (15)	−0.0029 (14)	−0.0101 (15)
O18	0.048 (2)	0.059 (2)	0.097 (3)	0.0194 (17)	0.0208 (19)	0.030 (2)
O19	0.0222 (12)	0.0239 (12)	0.0442 (15)	0.0033 (9)	−0.0041 (10)	−0.0090 (11)
O20	0.0369 (15)	0.0461 (17)	0.0446 (17)	−0.0025 (13)	0.0032 (13)	0.0001 (13)
O21	0.0448 (17)	0.0389 (16)	0.0519 (18)	0.0145 (13)	0.0113 (14)	0.0152 (14)
O22	0.0494 (18)	0.0419 (17)	0.0553 (19)	0.0009 (14)	−0.0129 (15)	0.0043 (14)
O23	0.0538 (19)	0.0310 (15)	0.060 (2)	0.0038 (13)	−0.0118 (15)	0.0063 (14)
O24	0.0309 (14)	0.0459 (16)	0.0350 (14)	−0.0020 (12)	−0.0001 (11)	0.0040 (12)
C1	0.0244 (15)	0.0106 (13)	0.0165 (14)	−0.0007 (11)	−0.0017 (11)	−0.0018 (10)
C2	0.0191 (14)	0.0169 (14)	0.0177 (14)	0.0021 (11)	−0.0016 (11)	−0.0033 (11)
C3	0.0202 (14)	0.0159 (14)	0.0169 (14)	0.0022 (11)	−0.0049 (11)	−0.0014 (11)
C4	0.0190 (14)	0.0136 (14)	0.0235 (15)	0.0044 (11)	−0.0052 (12)	−0.0035 (11)
C5	0.0163 (13)	0.0129 (13)	0.0158 (13)	0.0006 (10)	0.0001 (10)	0.0001 (10)
C6	0.0178 (13)	0.0163 (14)	0.0138 (13)	0.0027 (11)	0.0019 (11)	−0.0005 (11)
C7	0.0177 (13)	0.0124 (13)	0.0155 (13)	0.0017 (10)	0.0004 (11)	−0.0017 (10)
C8	0.0169 (13)	0.0129 (13)	0.0148 (13)	0.0016 (10)	−0.0012 (10)	0.0005 (10)
C9	0.0211 (14)	0.0165 (14)	0.0135 (13)	0.0032 (11)	−0.0002 (11)	−0.0039 (11)
C10	0.0184 (14)	0.0154 (14)	0.0145 (13)	0.0030 (11)	0.0008 (11)	−0.0036 (11)
C11	0.0253 (15)	0.0163 (14)	0.0105 (12)	−0.0035 (11)	0.0019 (11)	−0.0020 (11)
C12	0.0232 (15)	0.0181 (14)	0.0120 (13)	−0.0014 (11)	0.0019 (11)	−0.0021 (11)
C13	0.0221 (14)	0.0111 (13)	0.0085 (12)	0.0014 (10)	0.0012 (10)	0.0000 (10)
C14	0.0176 (14)	0.0203 (15)	0.0165 (14)	0.0012 (11)	0.0018 (11)	−0.0020 (11)
C15	0.0165 (13)	0.0136 (13)	0.0152 (13)	0.0001 (10)	0.0001 (11)	0.0020 (10)
C16	0.0216 (14)	0.0160 (14)	0.0118 (13)	0.0021 (11)	0.0019 (11)	−0.0008 (11)
C17	0.0255 (15)	0.0124 (13)	0.0151 (13)	0.0010 (11)	0.0017 (11)	−0.0022 (11)
C18	0.0151 (13)	0.0138 (13)	0.0134 (13)	−0.0005 (10)	0.0016 (10)	0.0028 (10)
C19	0.0186 (14)	0.0158 (14)	0.0114 (12)	0.0019 (11)	0.0014 (10)	−0.0003 (10)
C20	0.0227 (14)	0.0096 (13)	0.0164 (13)	0.0014 (11)	0.0003 (11)	−0.0025 (10)

### Geometric parameters (Å, º)

**Table:**

Cd1—O18	2.212 (3)	O15—Cd2^iv^	2.453 (2)
Cd1—O1	2.285 (2)	O16—C14	1.243 (4)
Cd1—O17	2.296 (3)	O16—Cd2^iv^	2.355 (3)
Cd1—O9	2.382 (2)	O17—H17B	0.8473
Cd1—O10	2.419 (2)	O17—H17C	0.8429
Cd1—O1^i^	2.457 (2)	O18—H18A	0.8500
Cd1—C11	2.733 (3)	O18—H18B	0.8500
Cd2—O5^ii^	2.268 (2)	O19—H19A	0.8500
Cd2—O19	2.277 (2)	O19—H19B	0.8500
Cd2—O7^iii^	2.282 (2)	O20—H20B	0.8500
Cd2—O13	2.316 (2)	O20—H20C	0.8500
Cd2—O16^iv^	2.355 (3)	O21—H21A	0.8500
Cd2—O15^iv^	2.453 (2)	O21—H21B	0.8500
Cd2—C14^iv^	2.737 (3)	O22—H22A	0.8500
Cd3—O20	2.217 (3)	O22—H22B	0.8500
Cd3—O14	2.224 (2)	O23—H23A	0.8500
Cd3—O12^v^	2.256 (2)	O23—H23B	0.8499
Cd3—O3^vi^	2.299 (2)	O24—H24A	0.8500
Cd3—O4^vi^	2.462 (2)	O24—H24B	0.8500
Cd3—O11^v^	2.585 (2)	C1—C5	1.511 (4)
Cd3—C2^vi^	2.721 (3)	C2—C7	1.504 (4)
O1—C1	1.282 (4)	C2—Cd3^vi^	2.721 (3)
O1—Cd1^i^	2.457 (2)	C3—C8	1.505 (4)
O2—C1	1.237 (4)	C4—C10	1.505 (4)
O2—H2	0.8199	C5—C6	1.391 (4)
O3—C2	1.251 (4)	C5—C10	1.406 (4)
O3—Cd3^vi^	2.299 (2)	C6—C7	1.400 (4)
O4—C2	1.259 (4)	C6—H6A	0.9300
O4—Cd3^vi^	2.462 (2)	C7—C8	1.391 (4)
O5—C3	1.262 (4)	C8—C9	1.395 (4)
O5—Cd2^vii^	2.268 (2)	C9—C10	1.390 (4)
O6—C3	1.242 (4)	C9—H9A	0.9300
O7—C4	1.266 (4)	C11—C15	1.505 (4)
O7—Cd2^iii^	2.282 (2)	C12—C16	1.492 (4)
O8—C4	1.248 (4)	C13—C18	1.515 (4)
O9—C11	1.260 (4)	C14—C19	1.505 (4)
O10—C11	1.252 (4)	C14—Cd2^iv^	2.737 (3)
O11—C12	1.247 (4)	C15—C20	1.389 (4)
O11—Cd3^v^	2.585 (2)	C15—C16	1.394 (4)
O11—H11	0.8193	C16—C17	1.394 (4)
O12—C12	1.262 (4)	C17—C18	1.389 (4)
O12—Cd3^v^	2.256 (2)	C17—H17A	0.9300
O13—C13	1.254 (4)	C18—C19	1.404 (4)
O14—C13	1.258 (4)	C19—C20	1.394 (4)
O15—C14	1.258 (4)	C20—H20A	0.9300
			
O18—Cd1—O1	114.51 (11)	H17B—O17—H17C	100.8
O18—Cd1—O17	105.77 (15)	Cd1—O18—H18A	114.7
O1—Cd1—O17	91.67 (10)	Cd1—O18—H18B	135.9
O18—Cd1—O9	152.67 (11)	H18A—O18—H18B	109.1
O1—Cd1—O9	89.85 (8)	Cd2—O19—H19A	107.1
O17—Cd1—O9	84.60 (10)	Cd2—O19—H19B	126.1
O18—Cd1—O10	99.67 (11)	H19A—O19—H19B	108.6
O1—Cd1—O10	144.38 (8)	Cd3—O20—H20B	105.3
O17—Cd1—O10	88.28 (10)	Cd3—O20—H20C	120.5
O9—Cd1—O10	54.67 (8)	H20B—O20—H20C	100.4
O18—Cd1—O1^i^	94.72 (13)	H21A—O21—H21B	100.1
O1—Cd1—O1^i^	77.86 (9)	H22A—O22—H22B	104.0
O17—Cd1—O1^i^	159.43 (10)	H23A—O23—H23B	109.7
O9—Cd1—O1^i^	77.79 (8)	H24A—O24—H24B	106.7
O10—Cd1—O1^i^	90.13 (8)	O2—C1—O1	123.9 (3)
O18—Cd1—C11	126.25 (11)	O2—C1—C5	117.6 (3)
O1—Cd1—C11	117.16 (9)	O1—C1—C5	118.3 (3)
O17—Cd1—C11	86.72 (10)	O3—C2—O4	121.8 (3)
O9—Cd1—C11	27.42 (8)	O3—C2—C7	118.2 (3)
O10—Cd1—C11	27.26 (8)	O4—C2—C7	119.9 (3)
O1^i^—Cd1—C11	82.55 (8)	O3—C2—Cd3^vi^	57.25 (16)
O5^ii^—Cd2—O19	164.72 (9)	O4—C2—Cd3^vi^	64.73 (16)
O5^ii^—Cd2—O7^iii^	89.68 (9)	C7—C2—Cd3^vi^	170.8 (2)
O19—Cd2—O7^iii^	85.15 (9)	O6—C3—O5	123.4 (3)
O5^ii^—Cd2—O13	86.30 (9)	O6—C3—C8	118.0 (3)
O19—Cd2—O13	88.23 (9)	O5—C3—C8	118.6 (3)
O7^iii^—Cd2—O13	139.19 (8)	O8—C4—O7	122.8 (3)
O5^ii^—Cd2—O16^iv^	92.31 (10)	O8—C4—C10	120.2 (3)
O19—Cd2—O16^iv^	101.56 (10)	O7—C4—C10	117.0 (3)
O7^iii^—Cd2—O16^iv^	85.25 (9)	C6—C5—C10	119.0 (3)
O13—Cd2—O16^iv^	135.45 (8)	C6—C5—C1	116.3 (3)
O5^ii^—Cd2—O15^iv^	82.85 (8)	C10—C5—C1	124.2 (3)
O19—Cd2—O15^iv^	110.44 (8)	C5—C6—C7	121.5 (3)
O7^iii^—Cd2—O15^iv^	138.17 (8)	C5—C6—H6A	119.3
O13—Cd2—O15^iv^	81.52 (8)	C7—C6—H6A	119.3
O16^iv^—Cd2—O15^iv^	54.23 (8)	C8—C7—C6	119.6 (3)
O5^ii^—Cd2—C14^iv^	86.15 (9)	C8—C7—C2	124.5 (3)
O19—Cd2—C14^iv^	109.13 (9)	C6—C7—C2	115.7 (3)
O7^iii^—Cd2—C14^iv^	111.38 (9)	C7—C8—C9	118.7 (3)
O13—Cd2—C14^iv^	108.83 (9)	C7—C8—C3	122.5 (3)
O16^iv^—Cd2—C14^iv^	26.93 (9)	C9—C8—C3	118.8 (3)
O15^iv^—Cd2—C14^iv^	27.35 (8)	C10—C9—C8	122.1 (3)
O20—Cd3—O14	109.96 (10)	C10—C9—H9A	118.9
O20—Cd3—O12^v^	104.98 (11)	C8—C9—H9A	118.9
O14—Cd3—O12^v^	118.76 (9)	C9—C10—C5	118.9 (3)
O20—Cd3—O3^vi^	128.71 (10)	C9—C10—C4	119.1 (3)
O14—Cd3—O3^vi^	106.24 (9)	C5—C10—C4	121.9 (3)
O12^v^—Cd3—O3^vi^	87.43 (9)	O10—C11—O9	122.8 (3)
O20—Cd3—O4^vi^	86.79 (10)	O10—C11—C15	119.2 (3)
O14—Cd3—O4^vi^	94.92 (8)	O9—C11—C15	117.8 (3)
O12^v^—Cd3—O4^vi^	136.12 (8)	O10—C11—Cd1	62.26 (16)
O3^vi^—Cd3—O4^vi^	54.72 (8)	O9—C11—Cd1	60.59 (16)
O20—Cd3—O11^v^	82.73 (10)	C15—C11—Cd1	172.2 (2)
O14—Cd3—O11^v^	83.20 (8)	O11—C12—O12	121.6 (3)
O12^v^—Cd3—O11^v^	53.26 (8)	O11—C12—C16	119.7 (3)
O3^vi^—Cd3—O11^v^	137.20 (8)	O12—C12—C16	118.8 (3)
O4^vi^—Cd3—O11^v^	167.97 (7)	O13—C13—O14	125.0 (3)
O20—Cd3—C2^vi^	108.01 (10)	O13—C13—C18	116.1 (3)
O14—Cd3—C2^vi^	103.16 (9)	O14—C13—C18	118.7 (2)
O12^v^—Cd3—C2^vi^	111.66 (9)	O16—C14—O15	122.6 (3)
O3^vi^—Cd3—C2^vi^	27.23 (9)	O16—C14—C19	118.7 (3)
O4^vi^—Cd3—C2^vi^	27.55 (8)	O15—C14—C19	118.7 (3)
O11^v^—Cd3—C2^vi^	164.19 (8)	O16—C14—Cd2^iv^	59.12 (17)
C1—O1—Cd1	127.97 (19)	O15—C14—Cd2^iv^	63.63 (16)
C1—O1—Cd1^i^	124.46 (19)	C19—C14—Cd2^iv^	176.1 (2)
Cd1—O1—Cd1^i^	102.14 (9)	C20—C15—C16	119.4 (3)
C1—O2—H2	109.5	C20—C15—C11	117.3 (3)
C2—O3—Cd3^vi^	95.52 (19)	C16—C15—C11	123.2 (3)
C2—O4—Cd3^vi^	87.72 (18)	C17—C16—C15	119.0 (3)
C3—O5—Cd2^vii^	133.2 (2)	C17—C16—C12	119.9 (3)
C4—O7—Cd2^iii^	103.09 (19)	C15—C16—C12	121.0 (3)
C11—O9—Cd1	91.98 (18)	C18—C17—C16	122.0 (3)
C11—O10—Cd1	90.47 (18)	C18—C17—H17A	119.0
C12—O11—Cd3^v^	85.03 (18)	C16—C17—H17A	119.0
C12—O11—H11	108.1	C17—C18—C19	118.8 (3)
Cd3^v^—O11—H11	132.4	C17—C18—C13	117.2 (3)
C12—O12—Cd3^v^	100.07 (19)	C19—C18—C13	124.1 (2)
C13—O13—Cd2	130.86 (19)	C20—C19—C18	119.3 (3)
C13—O14—Cd3	127.86 (18)	C20—C19—C14	117.8 (3)
C14—O15—Cd2^iv^	89.03 (18)	C18—C19—C14	122.9 (3)
C14—O16—Cd2^iv^	93.95 (19)	C15—C20—C19	121.5 (3)
Cd1—O17—H17B	112.3	C15—C20—H20A	119.2
Cd1—O17—H17C	110.8	C19—C20—H20A	119.2
			
O18—Cd1—O1—C1	64.7 (3)	C6—C5—C10—C4	173.8 (3)
O17—Cd1—O1—C1	−43.4 (3)	C1—C5—C10—C4	−14.6 (4)
O9—Cd1—O1—C1	−128.0 (2)	O8—C4—C10—C9	153.6 (3)
O10—Cd1—O1—C1	−132.8 (2)	O7—C4—C10—C9	−24.4 (4)
O1^i^—Cd1—O1—C1	154.4 (3)	O8—C4—C10—C5	−24.0 (4)
C11—Cd1—O1—C1	−130.6 (2)	O7—C4—C10—C5	158.0 (3)
O18—Cd1—O1—Cd1^i^	−89.68 (14)	Cd1—O10—C11—O9	2.9 (3)
O17—Cd1—O1—Cd1^i^	162.14 (11)	Cd1—O10—C11—C15	−171.3 (2)
O9—Cd1—O1—Cd1^i^	77.54 (9)	Cd1—O9—C11—O10	−3.0 (3)
O10—Cd1—O1—Cd1^i^	72.77 (15)	Cd1—O9—C11—C15	171.3 (2)
O1^i^—Cd1—O1—Cd1^i^	0.0	O18—Cd1—C11—O10	−14.6 (2)
C11—Cd1—O1—Cd1^i^	74.97 (11)	O1—Cd1—C11—O10	−177.20 (16)
O18—Cd1—O9—C11	−21.0 (4)	O17—Cd1—C11—O10	92.60 (19)
O1—Cd1—O9—C11	−175.02 (18)	O9—Cd1—C11—O10	177.2 (3)
O17—Cd1—O9—C11	93.29 (19)	O1^i^—Cd1—C11—O10	−104.99 (18)
O10—Cd1—O9—C11	1.57 (16)	O18—Cd1—C11—O9	168.2 (2)
O1^i^—Cd1—O9—C11	−97.41 (18)	O1—Cd1—C11—O9	5.6 (2)
O18—Cd1—O10—C11	168.1 (2)	O17—Cd1—C11—O9	−84.60 (19)
O1—Cd1—O10—C11	4.3 (2)	O10—Cd1—C11—O9	−177.2 (3)
O17—Cd1—O10—C11	−86.19 (19)	O1^i^—Cd1—C11—O9	77.82 (18)
O9—Cd1—O10—C11	−1.58 (16)	O18—Cd1—C11—C15	88.0 (16)
O1^i^—Cd1—O10—C11	73.30 (18)	O1—Cd1—C11—C15	−74.6 (16)
O5^ii^—Cd2—O13—C13	154.2 (3)	O17—Cd1—C11—C15	−164.8 (16)
O19—Cd2—O13—C13	−40.1 (3)	O9—Cd1—C11—C15	−80.2 (16)
O7^iii^—Cd2—O13—C13	−120.6 (3)	O10—Cd1—C11—C15	102.6 (16)
O16^iv^—Cd2—O13—C13	64.6 (3)	O1^i^—Cd1—C11—C15	−2.4 (16)
O15^iv^—Cd2—O13—C13	70.9 (3)	Cd3^v^—O11—C12—O12	2.5 (3)
C14^iv^—Cd2—O13—C13	69.5 (3)	Cd3^v^—O11—C12—C16	−178.1 (3)
O20—Cd3—O14—C13	−156.7 (2)	Cd3^v^—O12—C12—O11	−2.9 (4)
O12^v^—Cd3—O14—C13	−35.8 (3)	Cd3^v^—O12—C12—C16	177.7 (2)
O3^vi^—Cd3—O14—C13	60.3 (2)	Cd2—O13—C13—O14	7.2 (4)
O4^vi^—Cd3—O14—C13	115.0 (2)	Cd2—O13—C13—C18	−177.81 (18)
O11^v^—Cd3—O14—C13	−77.0 (2)	Cd3—O14—C13—O13	157.5 (2)
C2^vi^—Cd3—O14—C13	88.3 (2)	Cd3—O14—C13—C18	−17.3 (4)
Cd1—O1—C1—O2	163.6 (2)	Cd2^iv^—O16—C14—O15	5.0 (3)
Cd1^i^—O1—C1—O2	−47.2 (4)	Cd2^iv^—O16—C14—C19	−176.2 (2)
Cd1—O1—C1—C5	−21.1 (4)	Cd2^iv^—O15—C14—O16	−4.8 (3)
Cd1^i^—O1—C1—C5	128.2 (2)	Cd2^iv^—O15—C14—C19	176.4 (2)
Cd3^vi^—O3—C2—O4	5.3 (3)	O10—C11—C15—C20	73.4 (4)
Cd3^vi^—O3—C2—C7	−170.8 (2)	O9—C11—C15—C20	−101.0 (3)
Cd3^vi^—O4—C2—O3	−4.9 (3)	Cd1—C11—C15—C20	−25.0 (17)
Cd3^vi^—O4—C2—C7	171.1 (2)	O10—C11—C15—C16	−109.7 (3)
Cd2^vii^—O5—C3—O6	−94.5 (4)	O9—C11—C15—C16	75.8 (4)
Cd2^vii^—O5—C3—C8	86.6 (3)	Cd1—C11—C15—C16	151.9 (14)
Cd2^iii^—O7—C4—O8	3.3 (4)	C20—C15—C16—C17	1.2 (4)
Cd2^iii^—O7—C4—C10	−178.7 (2)	C11—C15—C16—C17	−175.6 (3)
O2—C1—C5—C6	116.5 (3)	C20—C15—C16—C12	−177.0 (3)
O1—C1—C5—C6	−59.1 (4)	C11—C15—C16—C12	6.2 (4)
O2—C1—C5—C10	−55.2 (4)	O11—C12—C16—C17	−153.6 (3)
O1—C1—C5—C10	129.1 (3)	O12—C12—C16—C17	25.8 (5)
C10—C5—C6—C7	3.8 (4)	O11—C12—C16—C15	24.6 (5)
C1—C5—C6—C7	−168.4 (3)	O12—C12—C16—C15	−155.9 (3)
C5—C6—C7—C8	−0.7 (4)	C15—C16—C17—C18	−1.2 (5)
C5—C6—C7—C2	174.6 (3)	C12—C16—C17—C18	177.0 (3)
O3—C2—C7—C8	−143.9 (3)	C16—C17—C18—C19	0.3 (5)
O4—C2—C7—C8	39.9 (4)	C16—C17—C18—C13	−179.2 (3)
Cd3^vi^—C2—C7—C8	158.4 (12)	O13—C13—C18—C17	−79.5 (3)
O3—C2—C7—C6	41.1 (4)	O14—C13—C18—C17	95.8 (3)
O4—C2—C7—C6	−135.1 (3)	O13—C13—C18—C19	101.0 (3)
Cd3^vi^—C2—C7—C6	−16.6 (15)	O14—C13—C18—C19	−83.7 (4)
C6—C7—C8—C9	−2.3 (4)	C17—C18—C19—C20	0.5 (4)
C2—C7—C8—C9	−177.2 (3)	C13—C18—C19—C20	−179.9 (3)
C6—C7—C8—C3	177.5 (3)	C17—C18—C19—C14	179.3 (3)
C2—C7—C8—C3	2.7 (5)	C13—C18—C19—C14	−1.2 (4)
O6—C3—C8—C7	−132.8 (3)	O16—C14—C19—C20	−11.6 (4)
O5—C3—C8—C7	46.2 (4)	O15—C14—C19—C20	167.3 (3)
O6—C3—C8—C9	47.1 (4)	Cd2^iv^—C14—C19—C20	−67 (3)
O5—C3—C8—C9	−133.9 (3)	O16—C14—C19—C18	169.6 (3)
C7—C8—C9—C10	2.3 (4)	O15—C14—C19—C18	−11.5 (4)
C3—C8—C9—C10	−177.5 (3)	Cd2^iv^—C14—C19—C18	114 (3)
C8—C9—C10—C5	0.8 (4)	C16—C15—C20—C19	−0.4 (4)
C8—C9—C10—C4	−176.9 (3)	C11—C15—C20—C19	176.6 (3)
C6—C5—C10—C9	−3.8 (4)	C18—C19—C20—C15	−0.5 (4)
C1—C5—C10—C9	167.7 (3)	C14—C19—C20—C15	−179.4 (3)

Symmetry codes: (i) −*x*, −*y*, −*z*; (ii) *x*, *y*+1, *z*−1; (iii) −*x*, −*y*+1, −*z*; (iv) −*x*+1, −*y*+1, −*z*−1; (v) −*x*+1, −*y*+1, −*z*; (vi) −*x*+1, −*y*, −*z*; (vii) *x*, *y*−1, *z*+1.

### Hydrogen-bond geometry (Å, º)

**Table:**

*D*—H···*A*	*D*—H	H···*A*	*D*···*A*	*D*—H···*A*
O11—H11···O22	0.82	2.16	2.895 (4)	149
O17—H17*C*···O8	0.84	2.32	3.158 (4)	179
O18—H18*B*···O3	0.85	2.50	3.292 (4)	156
O2—H2···O10^i^	0.82	2.53	3.237 (3)	145
O18—H18*A*···O10^vi^	0.85	2.19	3.031 (4)	168
O24—H24*B*···O15^v^	0.85	2.09	2.841 (4)	147
O19—H19*A*···O23^ii^	0.85	1.92	2.720 (4)	157
O17—H17*B*···O23^viii^	0.85	2.29	3.073 (5)	155
O19—H19*B*···O6^ix^	0.85	1.85	2.698 (3)	173
O20—H20*B*···O6^ix^	0.85	2.18	2.996 (4)	160
O20—H20*C*···O13^x^	0.85	2.23	3.047 (4)	161
O22—H22*A*···O2^x^	0.85	2.05	2.807 (4)	147
O23—H23*B*···O7^x^	0.85	2.09	2.894 (4)	158
O24—H24*A*···O4^xi^	0.85	1.94	2.783 (4)	173
O21—H21*A*···O9^xii^	0.85	1.91	2.748 (4)	168
O21—H21*B*···O12^xiii^	0.85	1.94	2.765 (4)	163
O22—H22*B*···O21^xiv^	0.85	1.99	2.825 (4)	166
O23—H23*A*···O2^xv^	0.85	2.20	2.944 (4)	147

Symmetry codes: (i) −*x*, −*y*, −*z*; (ii) *x*, *y*+1, *z*−1; (v) −*x*+1, −*y*+1, −*z*; (vi) −*x*+1, −*y*, −*z*; (viii) −*x*+1, −*y*, −*z*+1; (ix) *x*+1, *y*+1, *z*−1; (x) *x*+1, *y*, *z*; (xi) *x*+1, *y*+1, *z*; (xii) −*x*, −*y*+1, −*z*+1; (xiii) *x*, *y*, *z*+1; (xiv) −*x*+1, −*y*+1, −*z*+1; (xv) −*x*, −*y*, −*z*+1.

## References

[bb1] Lin, J.-D., Cheng, J.-W. & Du, S.-W. (2008). *Cryst. Growth Des.* **8**, 3345–3353.

[bb2] Prajapati, R., Mishra, L., Kimura, K. & Raghavaiah, P. (2009). *Polyhedron*, **28**, 600–608.

[bb3] Rigaku/MSC (2004). *CrystalClear* Rigaku/MSC Inc., The Woodlands, Texas, USA.

[bb4] Sheldrick, G. M. (2008). *Acta Cryst.* A**64**, 112–122. 10.1107/S0108767307043930 18156677

[bb5] Wang, X., Liu, Y., Xu, C., Guo, Q., Hou, H. & Fan, Y. (2012). *Cryst. Growth Des.* **12**, 2435–2444.

[bb6] Westrip, S. P. (2010). *J. Appl. Cryst.* **43**, 920–925.

